# Who likes meat, fish, and seafood? Influence of sex, age, body mass index, smoking, and olfactory efficiency on meat product preferences

**DOI:** 10.1002/fsn3.4275

**Published:** 2024-07-10

**Authors:** Magdalena Hartman‐Petrycka, Agata Lebiedowska, Magdalena Kamińska, Beata Krusiec‐Świdergoł, Barbara Błońska‐Fajfrowska, Joanna Witkoś, Sławomir Wilczyński

**Affiliations:** ^1^ Department of Basic Biomedical Science, Faculty of Pharmaceutical Sciences in Sosnowiec Medical University of Silesia Sosnowiec Poland; ^2^ Department of Physical Medicine, School of Health Sciences in Katowice Medical University of Silesia in Katowice Katowice Poland

**Keywords:** age, body mass index, fish, food preferences, meat, seafood, sex, smoking and smell

## Abstract

Meat, fish, and seafood are animal products that can be found in various forms in the human diet. In Western culture, there are trends to reduce meat consumption. This work was created to assess how various factors influence the fact that we like to eat meat, fish, and seafood. Exploring these relationships will contribute to our understanding of why dietary interventions for the above foods may be so difficult to implement in some groups of people. Two hundred eighty‐three people living in Poland took part in the study. An interview and olfactory tests were conducted together with assessments of food preferences from 25 types of food products. The extent to which sex, age, body mass index, tobacco addiction, and sense of smell influence ‘meat, fish and seafood’ consumption was assessed. Using the factor analysis, a coherent group of ‘meat, fish and seafood’ products was selected: beef, pork, veal, cured meats, poultry, fish dishes, and seafood. ‘Meat, fish and seafood’ was liked more by men, compared to women (*B* = .85; CI = .60, 1.10; *t* = 6.66, *η* ^2^ = .14; *p* < .001), whereas other factors did not affect the preference of dishes from this group as a whole. A detailed analysis of each type of food separately showed, however, that not only is gender important, but fish is liked more by older people, and cured meats by people who identify odors more efficiently. When planning a diet change, including the products mentioned above, we should consider the sex and age of the person to whom the diet is addressed, because men declare a higher derived pleasure from eating various types of meat, fish, and seafood than women, while older people like fish more.

## Graphical abstract

Meat, fish, and seafood are animal products that can be found in various forms in the human diet. In Western culture, there are trends to reduce meat excessive consumption. Our result shows. When planning a diet change, including the products mentioned above, we should consider the sex and age of the person to whom the diet is addressed, because men declare a higher derived pleasure from eating various types of meat, fish, and seafood than women, while older people like fish more.

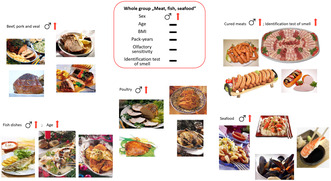

## 1 INTRODUCTION

There are many diets that provide all the nutrients in adequate portions. However, individual consumption varies greatly and is highly influenced by dietary trends and consumer preferences (Bender, 1997; Hartman‐Petrycka et al., 2022a). In recent years, one dietary trend that has been gaining in popularity is organic food consumption, which is directly linked to consumers’ growing awareness of the origin, provenance, and also composition of food, resulting in a conscious decision to refrain from consuming food of animal origin. Furthermore, the term ‘plant‐based diet’ colloquially referring to vegetarianism is used as an example of a healthy diet (Adamczyk & Maison, 2019; Cader & Lesiów, 2021).

The best known alternative diet, vegetarianism, is based mainly on the consumption of plant‐based foods such as vegetables, fruits, legumes, root and oleaginous crops, whole grains, nuts, mushrooms, and the simultaneous restriction or exclusion from the diet of meat, fish, and any products containing them. The decision to switch to a vegetarian diet can be dictated by ethical, religious, and cultural reasons. However, the most common reason for giving up meat consumption is concern for one's health (Adamczyk & Maison, 2019; Cader & Lesiów, 2021; Gadzala & Lesiów, 2019).

Depending on the range of products consumed, the concept of a vegetarian diet encompasses many versions, from radical to moderate. The most restrictive types include veganism (excludes all animal products, dairy, and eggs), witarianism (excludes all heat‐treated products), and fruitarianism (allows the consumption of vegetables and fruits that fall from the plant on their own, e.g., beans, tomatoes, and grapes). On the other hand, the more moderate versions include lacto‐vegetarianism (excludes meat products but allows the consumption of milk), lacto‐ovo‐vegetarianism (allows the consumption of plant products, eggs, and dairy products), ovo‐vegetarianism (allows the consumption of eggs but excludes dairy products) as well as lacto‐ovo‐pesco vegetarianism (allows the consumption of fish and seafood, dairy products, and eggs). Semi‐vegetarian diets include semi‐vegetarianism (a diet enriched with the consumption of eggs, dairy products, and small amounts of fish and poultry, but not including red meat) and pollo‐vegetarianism (including the consumption of poultry, excluding red meat products, fish, and seafood). A moderate form of vegetarianism is flexitarianism, which is characterized by a flexible approach depending on personal preferences or goals, and allows moderate consumption of different meats and meat products (Adamczyk & Maison, 2019; Derbyshire, 2017; Gadzala & Lesiów, 2019).

Nutritionally and medically, vegetarian diets can have both positive and negative health effects, which are largely determined by the type of diet. The benefits of vegetarian diets include low‐energy meals, a favorable sodium/potassium ratio, reduced intake of saturated fatty acids, low cholesterol, increased intake of fiber, vitamin C, and unsaturated fatty acids (Cader & Lesiów, 2021; Hargreaves et al., 2023). Additionally, a plant‐based diet is rich in complex carbohydrates, carotenoids, folic acid, and magnesium (Gadzala & Lesiów, 2019). These translate directly into health benefits by reducing the incidence of certain conditions such as cardiovascular disease (heart disease, hypertension, and stroke), gallstones, hypothyroidism, kidney stones, metabolic syndrome, obesity, and type 2 diabetes, as well as reducing the risk of cancer. A diet based mainly on plant products also improves the body's lipid metabolism, helps prevent bone mineral density disorders, and ensures better body regeneration and well‐being (Adamczyk & Maison, 2019; Cader & Lesiów, 2021). At the same time, despite its positive effects on bodily functions, a vegetarian diet that eliminates or limits the consumption of meat and meat products and fish can lead to deficiencies in high‐quality protein, long‐chain omega‐3 fatty acids, zinc, iron, calcium, vitamin B12, and vitamin D. Deficiencies or inadequate supplementation of the above nutrients can lead to reduced immunity, thyroid dysfunction, and even anemia (Adamczyk & Maison, 2019; Gadzala & Lesiów, 2019).

Due to current dietary patterns, contemporary attitudes toward meat are highly controversial. However, it should be emphasized that meat has a high nutritional value and is a natural complement to the nutrients that are deficient in a plant‐based diet and, most importantly, that, through evolution, the human species has adapted to meat consumption, since the anatomy of the digestive system exhibits features that lie somewhere between those of herbivores and carnivores (Baltic & Boskovic, 2015; Dal Bosco et al., 2022). Examples of physiological adaptations of the human body to meat consumption include the presence of a molecular mechanism that controls the absorption of heme in the small intestine, pancreatic enzymes responsible for fat digestion (lipase), and the presence of gene encoding proteins responsible for lipid transport (Ben‐Dor et al., 2021).

Today, consumers in highly developed countries can choose from a wide range of meats, the nutritional value of which can vary not only according to the type of meat, but also according to its origin and preparation. Beef (red meat), for example, has an impressive protein profile and the amino acids it contains are easily assimilated and play a key role during periods of rapid growth (childhood, pregnancy) and during aging by preventing muscle wasting. Beef also contains a wide range of minerals (zinc, iron, selenium, phosphorus, and potassium) and B vitamins. On the other hand, the high content of saturated fatty acids clearly indicates that the consumption of red meat, including beef, is associated with the development of cardiovascular disease (ischemic heart disease) (Baltic & Boskovic, 2015; Murimi, 2022). However, small portions of lean beef, roasted or cooked in some other way, combined with plenty of vegetables, fruit, whole grains, and physical activity can be part of a healthy diet (Sayer et al., 2017).

The most widely consumed meat in the world, for both nutritional and economic reasons, is poultry and especially chicken. The undoubted advantages of poultry over red meat are its significantly lower fat and cholesterol content and lower calorific value, especially when eaten without the skin. Poultry also contains bioactive compounds (taurine, glutathione), easily absorbed protein, and many micronutrients (Bordoni & Danesi, 2017; Cartoni Mancinelli et al., 1969; Dal Bosco et al., 2022). An interesting nutritional characteristic and important quality parameter of poultry meat is also its volume of n‐3 long‐chain polyunsaturated fatty acids (n‐3 LC‐PUFAs), which are deficient in current dietary patterns (Bordoni & Danesi, 2017; Cartoni Mancinelli et al., 1969).

Fish and seafood, on the other hand, are a group of products unequivocally associated with a healthy diet and are a rich source of n‐3 LC‐PUFAs, which improve cardiovascular function and reduce the risk of developing neurological diseases and allergies. In addition, their meat has a very high nutritional value due to its high quality and easily digestible proteins and particular micronutrients (iodine, fluorine) (Dal Bosco et al., 2022; Giorgia et al., 2023). Nevertheless, the consumption of fish and seafood is still low in regions with a rich food diversity (Bender, 1997). Enriching daily meals with fish and seafood would ensure a better dietary balance of polyunsaturated fatty acids, especially as the ratio of n‐6 to n‐3 fatty acids has increased to 20:1 in the last century, whereas it should be less than 4:1 (Cartoni Mancinelli et al., 1969).

Research by Derbyshire (2017) indicates that semi‐vegetarian and flexitarian diets have beneficial health effects by contributing to weight loss, improving metabolic function, and reducing the risk of developing diabetes and hypertension. Semi‐vegetarian diets may also be an acceptable form of vegetarianism for men who do not wish to completely exclude meat consumption (Derbyshire, 2017; Love & Sulikowski, 2018). In addition, based on current nutritional knowledge, moderate meat consumption is recommended for children, adolescents, and pregnant and lactating women, because it is a rich source of zinc, copper, easily absorbed heme iron and, above all, high‐value protein (Committee of Human Nutrition Science of Polish Academy of Sciences, 2019).

Olfactory perception plays an important role in food intake and digestion, e.g., through the mechanism of the cephalic phase of gastric juice secretion (Traczyk & Trzebski, 2020). In addition, olfactory dysfunction has been shown to be associated with an impaired perception of the taste of foods consumed (Zang et al., 2019). In the available literature, little attention has been paid to the role that olfactory sensitivity plays in shaping food preferences for meat, fish, and seafood dishes. For this reason, the aim of this study was to evaluate the influence of olfactory sensitivity on preferences for different meat, fish, and seafood products and to assess the impact of other personal factors, such as gender, age, body mass index, and level of tobacco addiction, on these food groups.

## 2 MATERIALS AND METHODS

The results presented in this paper are part of a larger research project. The first part of the results, analyzing the affect of different influences on the food preferences for products in Factor One group—unhealthy foods with a strong flavor—has already been published (Hartman‐Petrycka et al., 2022b). This study analyzes the effect of the same influences on the next food product group: Factor Two—meat products, fish, and seafood. As the data come from the same project as in the publication on unhealthy foods, the chapter on Materials and methods and some of the results are identical to those mentioned in the first publication in this series (Hartman‐Petrycka et al., 2022b).

### 2.1 Participants

The study involved 283 people aged between 18 and 82 years, living in Silesian Voivodeship Poland. The majority of the young people participating in the study were recruited among students of medical analytics, pharmacy, cosmetology, and biotechnology of the Medical University of Silesia. As the research was conducted within the Students' Science Club, a significant number of the participants were the students' family and friends. There were 190 women and 93 men, including 43 smokers, who participated in the study. The percentage of people belonging to each BMI (body mass index) category, according to WHO (World Health Organization) (2010), was as follows: 7.8% underweight (<18.5 kg/m^2^), 66.0% normal weight (18.5–24.9 kg/m^2^), 17.0% overweight (25.0–29.9 kg/m^2^), 7.8% first‐degree obesity (30.0–34.9 kg/m^2^), and 1.4% second‐degree obesity (35.0–39.9 kg/m^2^). Full characteristics of the respondents are summarized in Table 1.

**TABLE 1:** Characteristics of the study participants.

	*N*	Mean	Median	SD	Minimum	Maximum
Age (Years)	283	29.22	23.00	13.44	18.00	82.00
BMI	282	23.33	22.21	4.17	16.65	36.73
No. of years as a smoker	283	2.80	0.00	7.27	0.00	44.00
No. of cigarettes a day	283	2.27	0.00	5.91	0.00	50.00
Severity of addiction (pack‐years)	283	1.83	0.00	6.86	0.00	60.00
Olfactory sensitivity threshold (serial dilution)	283	7021	1024	15,208	0.00	65,636
Identification of smell	283	3.94	4.00	1.08	0.00	5.00

Abbreviations: BMI, body mass index; *N*, number of participants; SD, standard deviation.

In accordance with the Declaration of Helsinki, all subjects were informed about the purpose and method of the study and gave written consent to participate. The Bioethics Committee of the Silesian Medical University agreed to conduct the study (Resolution KNW/0022/KB1/47/12).

Exclusion criteria for the study were lack of nasal patency as demonstrated by a rhinomanometric test (total flow through the anterior nostrils less than 280 cm^3^), inability to understand the procedures during the study, and refusal to participate in the study. The exclusion of subjects from the study who did not have nasal patency was intended to eliminate such people in whom obstruction could cause short‐term olfactory impairment. Pregnant women did not participate in the study.

Preparation of the volunteers for participation in the olfactory tests: Each participant was asked to avoid foods and spices with a strong taste and smell, e.g., garlic, the day before the test, to take special care of their personal hygiene and not to use cosmetics with a strong smell. The study was conducted after a 30‐min acclimatization to the olfactometric laboratory conditions. During this time, no food or drink (other than still water), chewing gum, smoking, applying cosmetics, or engaging in physical exertion was allowed.

Determination of the olfactory sensitivity threshold: The olfactory sensitivity threshold to n‐butanol was assessed by an ECOMA T08 olfactometer using the dynamic olfactometry method in accordance with the guidelines issued by the Polish Committee for Standardization in accordance with European Standards PN‐EN 13725:2007 (Supplement S1). The olfactometer diluted n‐butanol at a concentration of 59.9 ppm (parts per million) with air and administered it to the participants' stations in the following dilution steps 2^16^, 2^15^, …, 2^3^, 2^2^. Odor samples alternated with air were sent to the test subjects' stations at a speed of 0.2 m/s, for 2.2 s. The participants' task was to press a button when they smelled an odor other than air. The measuring cycle was stopped when the odor substance was correctly indicated at least twice and no error was recorded if the air sample was selected. Before the test, the participants did not know the type of substance used. The assessment of the olfactory sensitivity threshold was carried out twice, the lower of the sensitivity thresholds obtained was chosen as the final result.

Identification test of smell: After a 15‐min break, the participants moved to a separate room where they were asked to judge which odor they could smell, based on the smell of a substance which had been applied to a smelling strip. Limonene, which smells like citrus, menthol, which smells like mint, phenethyl alcohol, which smells like flowers, eugenol, which smells like cloves, and n‐butanol, which smells like an alcoholic chemical, were used in the identification test. The respondents described the names of the odors with no further prompting; names similar to those presented above were accepted, e.g., limonene—lemon, lime, orange, citrus, and lemonade. The outcome of the trial was the number of correctly recognized odors.

Food preference test: The food preference test was conducted, prior to the olfactory test, during the acclimatization to the olfactometric laboratory conditions. The volunteers viewed a photo album with pictures of 24 types of food and sugary carbonated drinks (Supplement S2). They were asked to state how pleasant they found the food they were looking at. They marked their answer on 10 cm linear scales labeled at one end ‘0—not at all pleasant’ and at the other end ‘10—maximally pleasant’ (Supplement S3). The score was the distance from zero to the point marked by the subject. The types of food assessed were: fish dishes, egg dishes, sweet desserts, chocolate, sweets and jellybeans, crisps, dumplings, pasta, milk soup (this is a sweet dish made by pouring hot milk over food items, such as: boiled rice, pasta, oatmeal, chocolate chips or corn flakes, etc.), milk drinks, cheese, vegetables and salads, fruit, sausages and ham, beef and pork, poultry, bread, fast food, salty products, sour products, broth, soups, spicy dishes, seafood, and sugary carbonated drinks.

Statistical analysis: Statistical analysis was carried out using SPSS 21 software. A descriptive analysis was performed, then KMO (Kaiser–Meyer–Olkin) indices were checked for all the tested foods, Bartlett's test of sphericity was performed, and a factor analysis of the main components was carried out using VARIMAX rotation. After seven factors were identified, Factor Two was named ‘meat products, fish and seafood’ for the purposes of further analysis in the research. Regression models were built for the entire ‘meat products, fish and seafood’ group and for each component independently, i.e., beef, pork, and veal, cured meats, poultry, fish dishes, and seafood. The predictors in the regression models were sex, age, BMI, pack‐year, olfactory sensitivity, and odor identification. Nonstandardized regression coefficients (B) were given for numerical and dichotomous predictors, along with 95% confidence intervals (CI). A coefficient of multiple determination (R2) value was also provided for each analysis, in addition to effect–size ratios for each predictor; these were expressed as eta‐square. In the case of smoking, the indicator of the ‘pack‐years’ addiction (number of cigarette packs smoked per day times years of smoking) was used as a predictor.

## 3 RESULTS

**FIGURE 1:**
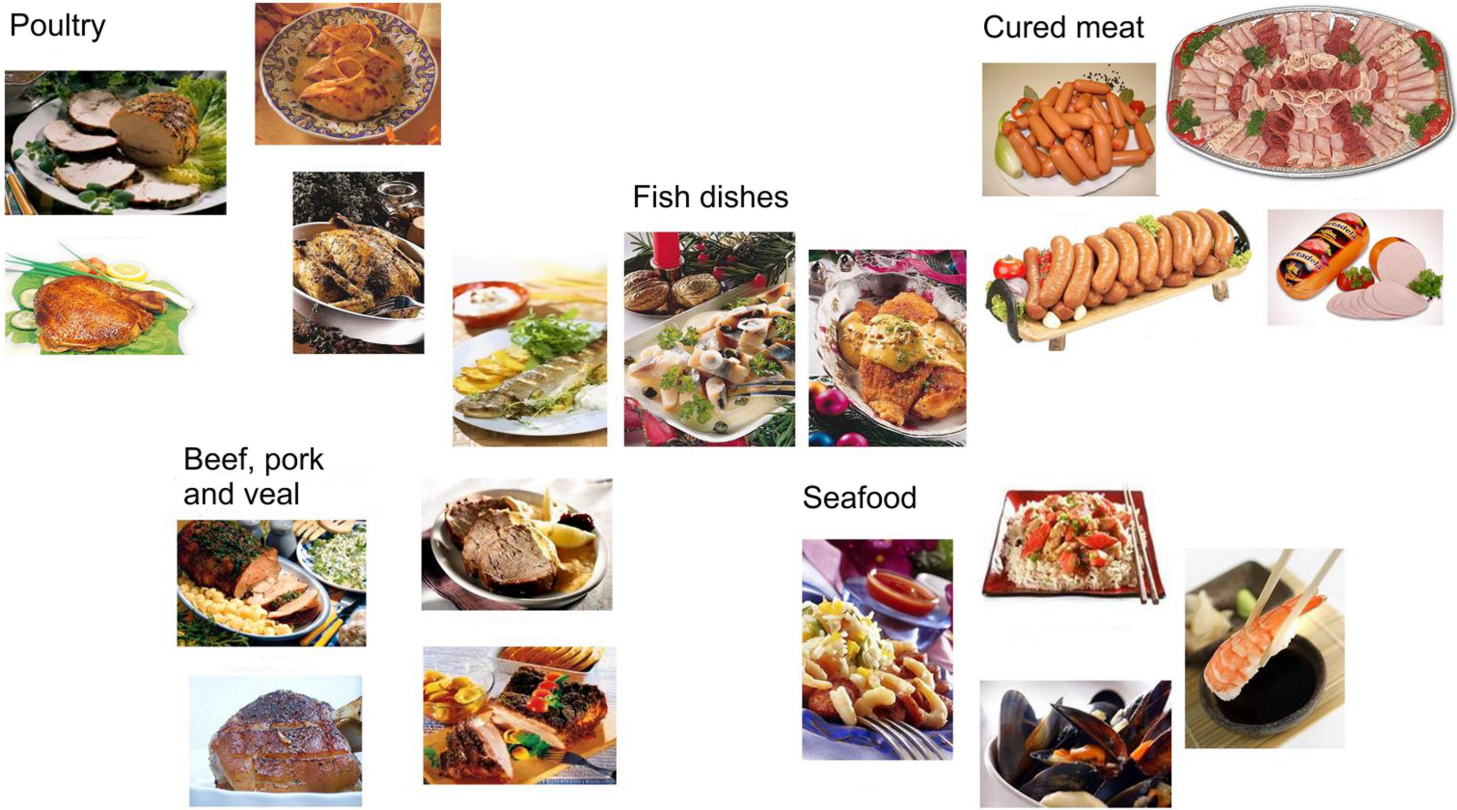
Foods that comprised Factor Two—‘meat, fish and seafood’.

A total of 25 types of food were analyzed in order to assess the preferences, so a factor analysis was performed using the principal component method with VARIMAX rotation. This technique allows a larger number of variables to be categorized into certain groups, such that the variables within each group relate to a similar factor. The KMO (Kaiser–Meyer–Olkin) value was 0.80, so it was acceptable (Zakrzewska, 1994). Thanks to Bartlett's test of sphericity, the hypothesis that the individual items are uncorrelated and that there was no factor structure among them (*χ* ^2^ = 2408.64, *df* = 300, *p* < .001) was rejected. Only factors for which the eigenvalue exceeded 1 were included. A clear factor solution was obtained. Overall, the seven factors identified explained a total of 62.10% of the variance for the scale items. Factor Two had an eigenvalue of 2.60 and explained 11.38% of the variance value. The factor loadings after VARIMAX rotation for Factor Two, named in this study as ‘meat, fish and seafood’, were beef, pork, and veal with values of 0.76, cured meats 0.73, poultry 0.70, fish dishes 0.53, and seafood 0.52. The foods that comprised Factor Two ‘meat, fish and seafood’ are shown in Figure 1 and the values of the declared pleasure of eating these foods are presented in Table 2.

**TABLE 2:** The values of the declared pleasure of eating various types of dishes, ranging from the most popular ones. Dishes marked in gray are the ones that have been identified by statistical analysis as a common ‘Factor Two—meat, fish and seafood’ group.

	*N*	Mean	Median	SD	Minimum	Maximum
Fruit	282	8.63	9.30	1.82	1.00	10.00
Desserts	283	8.24	9.50	2.47	0.00	10.00
Vegetables and salads	282	7.84	8.50	2.26	0.50	10.00
Poultry	282	7.69	8.25	2.29	0.00	10.00
Chocolate products	283	7.61	8.80	2.84	0.00	10.00
Bread	282	7.38	7.80	2.18	0.20	10.00
Pasta	282	7.05	7.60	2.45	0.00	10.00
Egg dishes	283	6.90	7.30	2.61	0.00	10.00
Flour‐based dishes	283	6.89	7.10	2.55	0.00	10.00
Soups	283	6.79	7.10	2.58	0.00	10.00
Broth	283	6.72	7.70	3.11	0.00	10.00
Cheeses	281	6.67	7.00	2.72	0.00	10.00
Cured meats	282	6.66	7.20	2.82	0.00	19.00
Fish dishes	283	6.66	7.00	2.67	0.00	10.00
Beef, pork, and veal	282	6.54	7.20	3.01	0.00	10.00
Sweets	282	6.24	6.90	3.20	0.00	10.00
Milk products	281	6.03	6.10	2.80	0.00	10.00
Sour products	282	6.00	6.00	2.88	0.00	10.00
Fast food	283	5.71	6.30	3.38	0.00	10.00
Crisps	283	5.58	6.00	3.16	0.00	10.00
Spicy dishes	283	5.47	5.50	3.30	0.00	10.00
Carbonated drinks	282	5.03	5.00	3.11	0.00	10.00
Salty snacks	283	4.84	5.00	2.93	0.00	10.00
Milk soup	282	3.52	2.90	3.14	0.00	10.00
Seafood	283	3.38	2.10	3.42	0.00	10.00

Abbreviations: *N*, number of participants; SD, standard deviation.

Factor Two ‘meat, fish and seafood’ became the dependent variable in the regression model (Table 3).

**TABLE 3:** The effect of predictors, such as sex, age, BMI, pack‐years, the olfactory sensitivity threshold to n‐butanol (serial dilution), and identification test of smell on the declared pleasure of the group of dishes selected in the factor analysis as Factor Two ‘meat, fish and seafood’.

Dependent variables	$R_{c}^{2}$	Predictors	*B*	CI		*t*	*η* ^2^	*p*
Factor Two meat, fish, and seafood	.16	Constant	−1.53	−2.39	−.66	−3.48	.04	.001
		Sex	.85	.60	1.10	6.66	.14	<.001
		Age	<.01	−.01	.01	.41	<.01	.680
		BMI	<.01	−.03	.03	.11	<.01	.914
		Pack‐years	−.01	−.02	.01	−.55	<.01	.586
		Olfactory sensitivity threshold to n‐butanol (serial dilution)	<.01	<.01	<.01	−.69	<.01	.489
		Identification test of smell	.08	−.03	.19	1.44	.01	.152

Abbreviations: B, unstandardized regression coefficients; *p*, level of significance; CI, confidence interval; $R_{c}^{2}$, multiple determination coefficient; *t*, *t* statistic; *η* ^2^, effect size.

**FIGURE 2:**
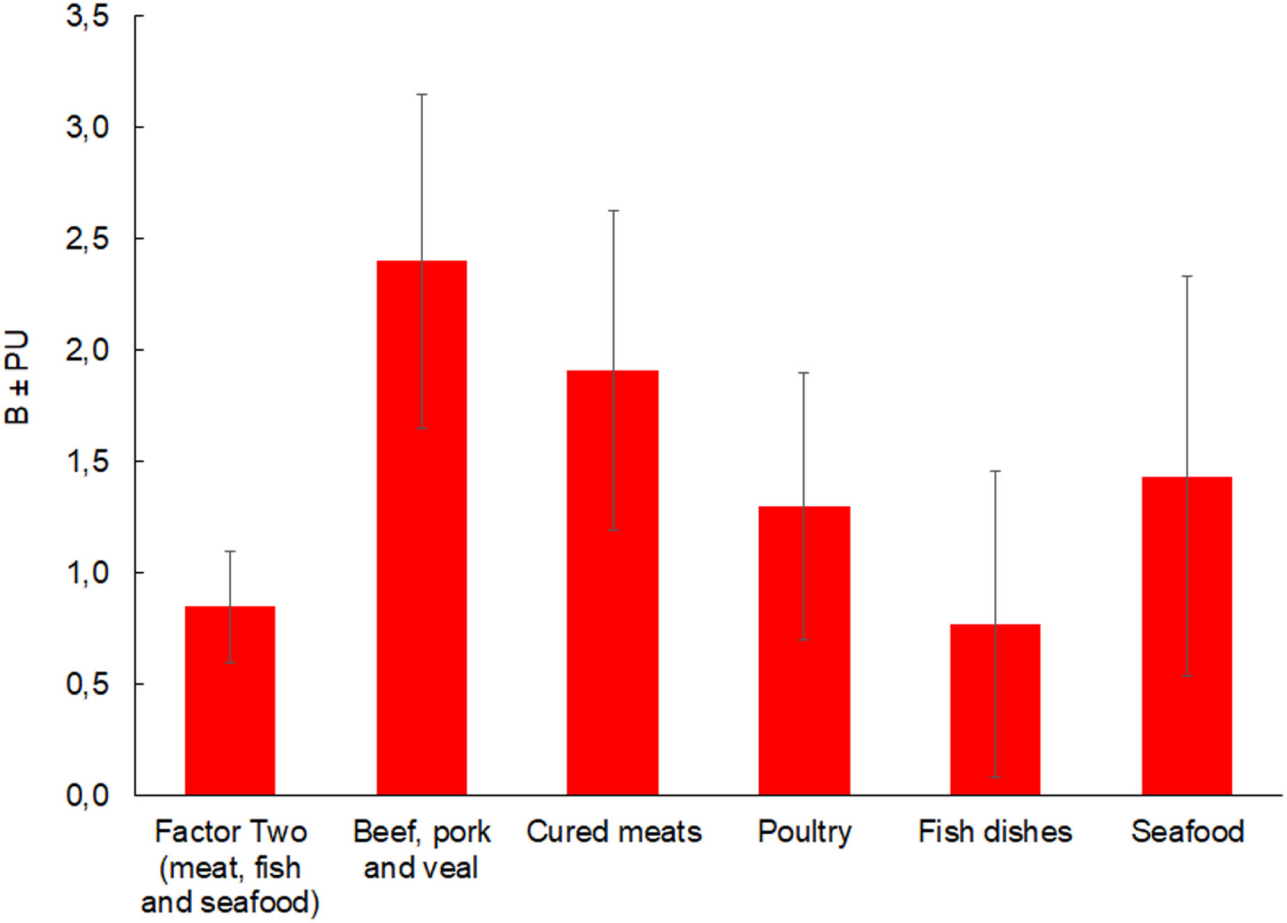
The influence of male gender on food preferences. B, Unstandardized regression coefficients; *p*, level of significance; CI, confidence interval; $R_{c}^{2}$, multiple determination coefficient; *t*, *t* statistic; *η* ^2^, effect size.

The value of the multiple coefficient of determination in this model is $R_{c}^{2}$ = .16. Sex had the greatest impact on the increased preference for Factor Two, where men are more likely than women to declare that they like ‘meat, fish and seafood’ products (*B* = .85; CI = .60, 1.10; *t* = 6.66, *η* ^2^ = .14; *p* < .001) (Figure 2). The other predictors do not have a statistically significant effect on the whole group of ‘meat, fish and seafood’ products.

From the analysis of the influence of factors on the particular type of food categorized as ‘meat, fish and seafood’, sex was seen as a significant effect for all products in this group (Table 4).

**TABLE 4:** Influence of predictors, such as sex, age, BMI, pack‐years, olfactory sensitivity threshold to n‐butanol (serial dilution), and dentification test of smell on the declared pleasure of individual dishes, identified in the factor analysis as Factor Two ‘meat, fish and seafood’.

Dependent variables	$R_{c}^{2}$	Predictors	*B*	CI		*t*	*η* ^2^	*p*
Beef, pork, and veal	.15	Constant	2.08	−.51	4.68	1.58	.01	.115
		Sex	2.40	1.65	3.15	6.28	.13	<.001
		Age	.01	−.02	.05	.82	<.01	.412
		BMI	.01	−.08	.11	.24	<.01	.809
		Pack‐years	<.01	−.05	.06	.04	<.01	.968
		olfactory sensitivity threshold to n‐butanol (serial dilution)	<.01	<.01	<.01	.75	<.01	.456
		Identification test of smell	.13	−.20	.46	.80	<.01	.425
Cured meats	.11	Constant	2.37	−.11	4.86	1.88	.01	.061
		Sex	1.91	1.19	2.63	5.22	.09	<.001
		Age	.01	−.02	.04	.66	<.01	.511
		BMI	.01	−.08	.10	.15	<.01	.877
		Pack‐years	−.01	−.06	.04	−.33	<.01	.739
		Olfactory sensitivity threshold	<.01	<.01	<.01	.13	<.01	.896
		Identification test of smell	.32	.01	.64	2.01	.01	.045
Poultry	.07	Constant	5.99	3.93	8.05	5.72	.11	<.001
		Sex	1.30	.70	1.90	4.29	.06	<.001
		Age	.01	−.02	.04	.73	<.01	.467
		BMI	−.04	−.11	.04	−.94	<.01	.347
		Pack‐years	.01	−.04	.05	.25	<.01	.804
		Olfactory sensitivity threshold	<.01	<.01	<.01	−.24	<.01	.812
		Identification test of smell	.14	−.13	.40	1.02	<.01	.310
Fish dishes	.08	Constant	3.92	1.53	6.31	3.23	.04	.001
		Sex	.77	.08	1.46	2.18	.02	.030
		Age	.05	.02	.08	3.58	.04	<.001
		BMI	−.02	−.11	.07	−.43	<.01	.670
		Pack‐years	−.03	−.08	.03	−.99	<.01	.324
		Olfactory sensitivity threshold	<.01	<.01	<.01	−.99	<.01	.323
		Identification test of smell	.17	−.13	.48	1.13	<.01	.261
Seafood	.06	Constant	1.31	−1.77	4.40	.84	<.01	.403
		Sex	1.43	.54	2.33	3.15	.03	.002
		Age	−.03	−.06	.01	−1.32	.01	.189
		BMI	.01	−.10	.12	.19	<.01	.849
		Pack‐years	−.04	−.10	.03	−1.09	<.01	.278
		Olfactory sensitivity threshold	<.01	<.01	<.01	−.98	<.01	.328
		Identification test of smell	.21	−.19	.60	1.03	.00	.306

Abbreviations: B, unstandardized regression coefficients; *p*, level of significance; CI, confidence interval; $R_{c}^{2}$, multiple determination coefficient; *t*, *t* statistic; *η* ^2^, effect size.

Compared with women, men reported greater pleasure in eating beef, pork, and veal (*B* = 2.40; CI = 1.65, 3.15; *t* = 6.28, *η* ^2^ = .13; *p* < .001), cured meats (*B* = 1.91; CI = 1.19, 2.63; *t* = 5.22, *η* ^2^ = .09; *p* < .001), poultry (*B* = 1.30; CI = .70, 1.90; *t* = 4.29, *η* ^2^ = .06; *p* < .001), fish dishes (*B* = .77; CI = .08, 1.46; *t* = 2.18, *η* ^2^ = .02; *p* = .030), and seafood (*B* = 1.43; CI = .54, 2.33; *t* = 3.15, *η* ^2^ = .03; *p* = .002) (Figure 2.).

Not only is gender important, because older people liked fish more (*B* = .05; CI = .02, .08; *t* = 3.58, *η* ^2^ = .04; *p* < .001), but also people who identify odors more efficiently liked cured meats more (*B* = .32; CI = .01, .64; *t* = 2.01, *η* ^2^ = .01; *p* = .045) (Table 4).

## 4 DISCUSSION

In the present study, thanks to the VARIMAX factor analysis, a group of dishes was selected from the 25 types of food, for which the food preferences of the respondents were convergent. The most convergent results were the preferences for the following foods: beef, pork, veal, poultry, cured meats, fish dishes, and seafood. In the research, these foods form a group called ‘meat, fish and seafood’. All the products in this group are of animal origin. What they all have in common are their natural origin and their high nutritional value, especially their high protein content. The predictors taken into account that could influence the preferences for the selected group of foods were age, gender, BMI, number of cigarettes smoked, and olfactory performance, which were measured by two types of tests.

Of all the predictors analyzed in this study, the gender of the participants had the greatest impact on their preference for the entire group of ‘meat, fish and seafood’ products, defined as Factor Two. The regression model showed that the men liked the foods included in this group more than the women. This observation is confirmed by numerous scientific reports. According to research conducted in the USA, men have a higher number of behaviors defined as risky and harmful to health compared to women, as well as a lower number of health‐promoting behaviors. In comparison with women, men consumed more animal products than recommended by nutrition experts. The difference between the sexes was particularly pronounced with regard to meat intake. Men ate more meat of all types; in addition, more than half of this amount was red and processed meat (RPM), considered the least healthy type (Nakagawa & Hart, 2019; U.S. Department of Health and Human Services and U.S. Department of Agriculture, 2015; Wang et al., 2010).

Based on an analysis of 3 years of data from the National Diet and Nutrition Survey (NDNS), Maguire and Monsivais (2015) reported a higher RPM intake in men compared to women. The same results were found by Clonan et al. (2015), who investigated RPM intake based on a postal survey of consumers, and by Meier and Christen (2012), who analyzed data on food production and consumption in Germany.

The difference in meat consumption between men and women can be explained by differences in food preferences, which strongly influence the consumption of a particular type of food. A study by Caine‐Bish and Scheule (2009) examined the food preferences of primary, middle, and high school students. Boys were significantly more likely than girls to select the category ‘beef, pork, barbecue (BBQ) and fish’, suggesting that gender differences in preferences exist even among schoolchildren and adolescents.

Lombardo et al. (2020) studied gender‐dependent differences in relation to dietary habits and preferences. Women were more likely to indicate whole‐grain products and vegetables, while men were significantly more likely to indicate animal products: eggs and RPM.

These aforementioned reports confirm that men tend to eat more meat, especially red meat, than women. Although this phenomenon is present in the public consciousness, as evidenced by advertising campaigns targeted at men (Castonguay & Bakir, 2018), the mechanisms explaining it have not yet been identified.

Ritzel and Mann (2021) hypothesized that gender‐specific biological factors may account for the differences seen in meat consumption. In their study, they used data from the US National Health and Nutrition Examination Survey (NHANES) on the consumption of all meats and red meat for men and women in seven age groups. A slight discrepancy could already be observed in childhood (5–11 years), while during adolescence (12–20 years) and early adulthood (21–35 years) the difference in the amount of meat consumed between men and women increased markedly, reaching a maximum between the ages of 51 and 65 years. In the later stages of life (65–80 years), there was a significant decrease in the difference in meat consumption between the sexes. According to the authors, if the observed difference in meat consumption between men and women were biologically determined, the value would remain constant throughout a person's life. This study suggests a link between the amount of meat consumed and the existence of masculine traits.

It cannot be ruled out that differences in meat preferences and consumption result from the level of sex hormones, especially testosterone (Fantus et al., 2020; Pohlmann, 2014), but so far no conclusive evidence has been provided to support this thesis.

The differences in dietary choices between men and women may be due to psychological and sociocultural factors. Studies using the Implicit Association Test (IAT) have found strong implicit associations between ‘meat’ and ‘healthy’, ‘meat’ and ‘men’, and ‘meat’ and ‘masculinity’ in both sexes (Love & Sulikowski, 2018). It is worth noting that the strength of the association was greater for men than for women. Another social psychology study suggests a strong association between meat and strength and male sexuality (Nath, 2010). Meat meals, which are considered highly nutritious and filling, are associated with men who do physical work; for many men, a meal without meat is considered neither healthy nor appropriate (Sobal, 2005; Thomas, 2016). Furthermore, the exclusion of animal products for men on a vegan diet is seen as less masculine (Bogueva et al., 2020; Thomas, 2016). The strong association, in Western societies, between meat and men and their masculine identity is probably the main reason why men are so reluctant to limit their meat intake.

Women and men justify the presence of meat in their diet differently. Men are more likely to have direct pro‐meat attitudes and rationalize meat consumption as stated by the ‘*Three N's of Justification’* rule (Joy & Dogs, 2010). In accordance with this, eating meat is natural, normal, and necessary. Piazza et al. (2015) proposed the ‘*Four N's of justification*’ rule, complementing the previous one with the argument, chosen equally often by men, that eating meat is ‘nice’. Women are more likely to use indirect strategies to justify eating meat, such as not associating live animals with food and not dwelling on the killing of animals (Rothgerber, 2013).

Women are more likely than men to express disgust and show a reluctance to eat meat. They are more motivated in their food choices by animal and environmental welfare (Rothgerber, 2013). Research in Poland and worldwide confirms that women predominate among ethically motivated vegetarians (Adamczyk & Maison, 2019; Lombardo et al., 2020). The probable reason for this gender difference is women's higher level of empathy toward animals, as well as the Machiavellianism found more often in the male gender (Mertens et al., 2020).

Women show more interest in the topic of healthy eating, current recommendations, and expert guidelines for a good diet. As a result, they have more knowledge in this area than men. The desire to live up to the social norms of Western culture regarding a healthy body weight and a slim figure is also important (Górecka et al., 2009; Kamińska & Hartman‐Petrycka, 2021; Lombardo et al., 2020).

The preference for fish dishes and the frequency of fish consumption depend mainly on the geographic location of the consumers as well as on many sociological and cultural factors. Wennberg et al. (2012), who conducted a study in northern Sweden, showed an association between fish consumption and a healthy lifestyle (e.g., fruit and vegetable consumption, physical activity) in both sexes. Fish consumption did not differ significantly between men and women. However, it is important to consider that the region has a cultural association with fishing, which may have influenced the inhabitants’ greater awareness of a healthy diet. In a Belgian study (Verbeke et al., 2007), men consumed less fish and had less knowledge about its health‐promoting properties than women, which is in line with research conducted in Poland among residents of the Silesian Voivodeship (Kamińska & Hartman‐Petrycka, 2021). In a study conducted in Bangladesh, however, men consumed twice as much fish as women (Rahman & Islam, 2020).

From a gender perspective, seafood can be associated with both feminine and masculine characteristics. As a food, it is considered more feminine because of its lightness and the way it is eaten. On the other hand, seafood, like fish, can be associated with masculine occupational and recreational activities such as fishing (Rozin et al., 2012; Wien et al., 2021). In a Norwegian study (Wien et al., 2021), seafood preparation was perceived by men as one of their male culinary skills and they also consumed more of it than women. Seafood is often seen as a difficult dish to prepare, allowing men to take up the challenge and demonstrate their culinary skills.

The so‐called hunting hypothesis may be used in an attempt to explain the reason for the stronger male preference for meat, fish, and seafood in this study (Lee & DeVore, 2017; Wężowicz‐Ziółkowska, 2018). Hunting, especially the need to obtain food by attacking, and the greater female approbation for food‐providing males, may have had a decisive influence on human evolution. Natural selection over two million years may have led to the development of a predatory instinct in humans, stronger in males, which was essential for the survival of the species. It is therefore possible that food preferences are evolutionarily determined.

In the present study, statistical analysis showed that all types of products included in the category ‘meat, fish and seafood’ are more preferred by men. This preference based on sex is the strongest for red meat (beef, pork, and veal), explaining 13% of the variation in preference for this type of meat. A strong relationship was also found for cured meats, explaining 9% of the variation in preference, and poultry meat, explaining 6% of the variation. Additional correlations were also observed. Fish was more preferred by older people and this observation was supported by the studies of Wennberg et al. (2012) and Verbeke et al. (2007), where low fish consumption among young people was associated with an insufficient knowledge of its nutritional value. Fish meat is considered to be easily digestible due to its lack of connective tissue, but the presence of bones, characteristic odor, taste, and texture may negatively affect its consumption (Birch et al., 2018). In a study conducted among residents of the Silesian Voivodeship in Poland, fish consumption increased with the age of the subjects. It cannot be ruled out that the prophylactic administration of fish oil in schools in the 1960s and 1970s in Poland may have influenced the results of the study (Kamińska & Hartman‐Petrycka, 2021).

Another additional relationship observed in the study was a greater preference for cured meats among those with a better ability to recognize odors, as confirmed by the odor identification test. The second olfactory sensitivity threshold test, however, did not confirm such a relationship. As a biological stimulator of food intake, smell plays an important role in the cephalic phase of food digestion. In a study by Ramaekers et al. (2014), a one‐minute exposure to the sight and smell of pizza increased appetite for this type of meal more than for other foods. The results of the present study, however, indicate that in the case of food preference, its contribution is not as important as it might have been predicted.

### 4.1 Limitations

The study presented here is cross‐sectional in nature, which imposes a number of limitations on its interpretation. As this type of study involves a single measurement, a cause‐and‐effect relationship cannot definitely be established, unless it is clearly based on physiology. This type of study can also be affected by systematic error and confounding variables, as well as by the selection method used for the study group. A relatively large number of people participated in this study, but it did not constitute a representative group for the Polish population. As described in the methodology, a large number of medical students took part in the study and, therefore, the study group contained a higher number of women, a lower mean age value, a lower percentage of overweight and obese people, and was characterized by a preference for healthier foods due to a higher level of knowledge, compared to the general Polish population. Thus, the study did not aim to identify the most preferred type of food from the list presented in Table 2 for the Polish population. Its main objective was to find a relationship between factors characterizing the subjects and their food preferences.

Dietary history was not collected in the study. Food preferences were assessed on the basis of the subjects’ reported enjoyment of eating a particular type of food. Subjects’ food preferences were not indicative of the actual consumption of meat, fish, and seafood in their diet. The subjects’ self‐reported food preferences may be subject to social approval bias, resulting from a desire to conform to social expectations in their answers and to present themselves in a better light to the interviewer. An example of the influence of the social expectation effect on the outcome of a survey can be found in the work of Rothgerber (2013), in which women underreported the amount of meat they consumed when also asked to provide arguments to justify their consumption. In this case, the women's reported meat consumption was lower than that of a group of women who were not exposed to information about animal suffering and stress. In order to limit the influence of social expectations on the results of this study, the questions included in the questionnaire asked how much the respondent liked the product, but did not ask how often it was consumed. In addition, the anonymous questionnaires were completed by the subjects themselves to avoid the discomfort of direct supervision by the researcher.

## 5 CONCLUSIONS

Being male is an important factor influencing the higher declared preference for the whole group of ‘meat, fish and seafood’ foods and for each individual type of food in this group, i.e., beef, pork, and veal, cured meats, poultry, fish dishes, and seafood. This finding, which is consistent with literature data, suggests that it would be beneficial to identify the reason for men's strong preference for meat foods, which would increase the effectiveness of measures to reduce meat consumption, especially red meat, and to choose a more balanced diet.

Older people's reported enjoyment of fish consumption was shown to be higher, which is a positive development, given the value of fish as a food and that it may contribute to maintaining health in old age.

The sense of smell does not play a major role in the preference for ‘meat, fish and seafood’ dishes. Of all the foods in this group, only cured meat was preferred by those with a better sense of smell.

There was no correlation between BMI and smoking severity and the declared intake of the entire ‘meat, fish and seafood’ group of foods or of each individual type of food in this group separately.

## AUTHOR CONTRIBUTIONS

**Magdalena Hartman‐Petrycka:** Conceptualization (equal); formal analysis (equal); investigation (equal); methodology (equal); validation (equal); visualization (equal); writing – review and editing (equal). **Agata Lebiedowska:** Investigation (equal); methodology (equal); writing – review and editing (equal). **Magdalena Kamińska:** Conceptualization (equal); investigation (equal); writing – original draft (equal). **Beata Krusiec‐Świdergoł:** Conceptualization (equal); investigation (equal); writing – original draft (equal). **Barbara Błońska‐Fajfrowska:** Formal analysis (equal); methodology (equal); resources (equal); supervision (equal); visualization (equal). **Joanna Witkoś:** Formal analysis (equal); resources (equal); writing – review and editing (equal). **Sławomir Wilczyński:** Conceptualization (equal); resources (equal); software (equal); supervision (equal); writing – review and editing (equal).

## CONFLICT OF INTEREST STATEMENT

The authors declare no conflicts of interest.

## Supporting information


Supplement S1.

## Data Availability

The data that support the findings of this study are available on request from the corresponding author.
