# Supplementary material for: Who likes meat, fish, and seafood? Influence of sex, age, body mass index, smoking, and olfactory efficiency on meat product preferences
Source: Food Sci Nutr. 2024 Jul 10;12(9):6799–809. doi: 10.1002/fsn3.4275 (PMC11561781; doi:10.1002/fsn3.4275)
Supplement: Supplementary file 1 — Supplement S1. [file FSN3-12-6799-s001.docx]

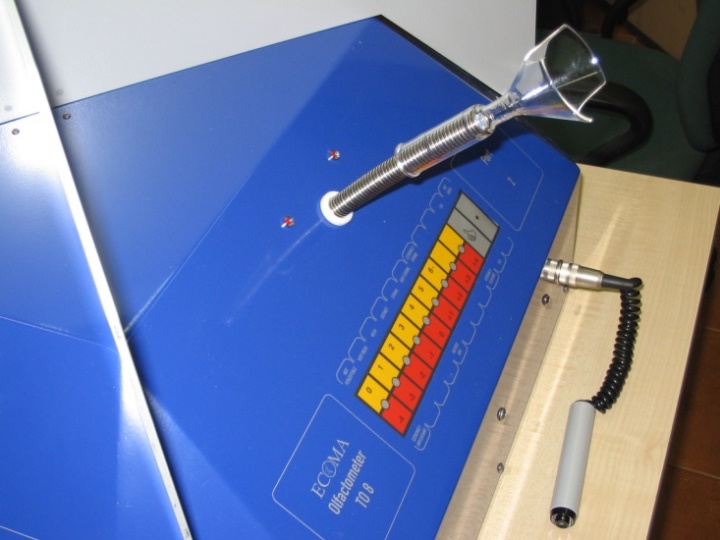


**Suplement S1.** Measuring station in T08 olfactometer


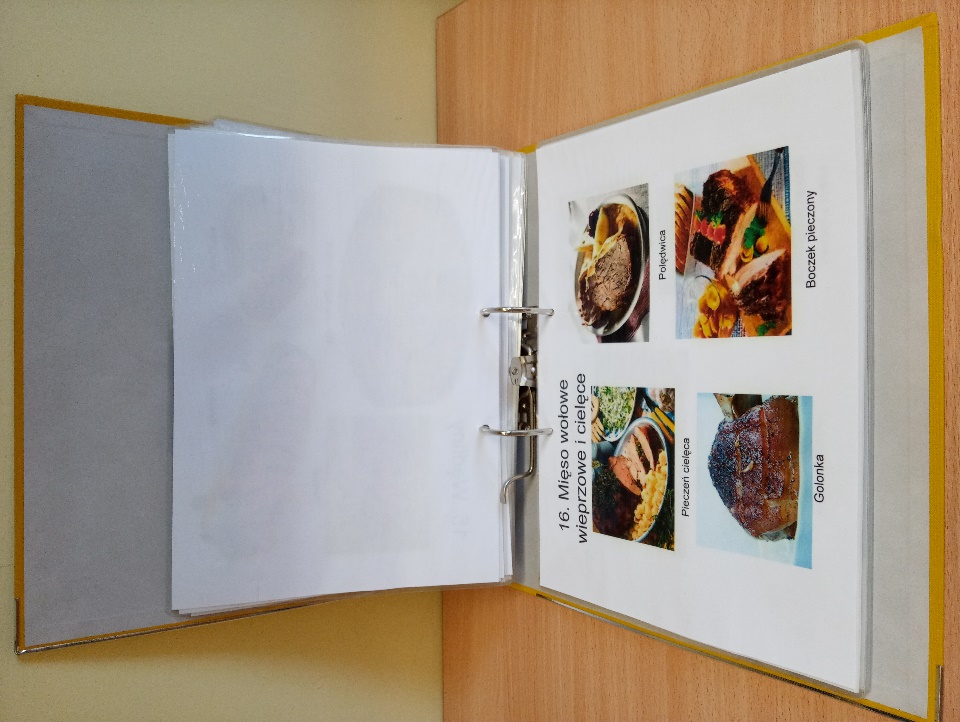


**Supplement S2.** Photo album with pictures of twenty-four types of food and sugary carbonated drinks


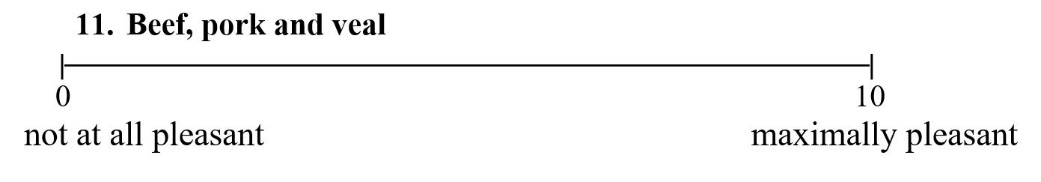


**Supplement S3.** Linear scales for food preferences
